# Pediatric Open Globe Injury in Tertiary Hospitals of Khobar and Dhahran

**DOI:** 10.7759/cureus.21284

**Published:** 2022-01-16

**Authors:** Faten A Al Majed, Fatemah T Al Shamlan, Mohammed A Alawazem, Hussain H Alsadah, Hossain S Al Beshri, Mohammed A Al Abdulwahhab

**Affiliations:** 1 Ophthalmology, King Fahd Hospital of the University, Khobar, SAU; 2 College of Medicine, Imam Abdulrahman Bin Faisal University, Dammam, SAU; 3 Ophthalmology, Dhahran Eye Specialist Hospital, Dhahran, SAU

**Keywords:** eye trauma, rupture globe, eye injury, pediatric, open globe injury

## Abstract

Background

Every year, 3.3 to 5.7 million eye injuries occur worldwide in children. Open globe injury is a common type of trauma that leads to blindness in all age groups. This study aimed to review and discuss the epidemiology, causes, and clinical outcome of pediatric open globe injury.

Methods

A retrospective chart review of all pediatric open globe injuries presented to King Fahad Hospital of the University and Dhahran Eye Specialist Hospital was conducted between 2010 and 2020. Data analyses were done to identify factors predicting ultimate visual acuity.

Results

The overall number of cases was 502, of which 120 cases were children and of the documented 118 cases, around 82 (69.5%) were males with an average age of 5.1 years. The traumatic object was sharp in 90 (89.1%) cases. The most common object was glass, presented in 18 (18.4%) cases. Most injuries were accidental (68.9%) and 48 (71.6%) cases out of 67 occurred inside the house. The factors associated with better visual outcome (20/40 or better) were sharp injuries, absence of hyphema, absence of vitreous hemorrhage, trauma with intraocular foreign body, undergone one operation, and absence of cataract at presentation.

Conclusion

The ultimate visual outcome post open globe injury could be predicted from the severity of the presenting signs. Also, the household environment carries the highest risk, hence it is not always a safe haven for children. Thus, childproofing the house and adult supervision is advisable.

## Introduction

Trauma to the eye especially open globe injury is a frequent cause of blindness in children. Approximately 8-14% of all children's injuries are eye injuries. Open globe injury accounts for 28.9-49.7% of all eye injuries [1,2]. This type of injury is considered a significant cause of hospitalization of children and might lead to visual impairments. Open-globe injury costs more than any other type of pediatric eye injury. It costs more than $88 million annually [3,4]. Thus, this study aimed to investigate and discuss the epidemiology, causes, and outcomes of pediatric open globe injury in the eastern province of Saudi Arabia and to identify its prevention methods.

## Materials and methods

A retrospective chart review of all cases of open globe injuries was conducted including patients aged 12 or younger, who presented to King Fahd Hospital of the University and Dhahran Eye Specialist Hospital from January 2010 to December 2020.

The data collection included demographics, age, gender, the mechanism of injury (blunt or penetrating), the object causing the injury, manner of injury (accidental or purposeful), the place where the injury occurred, time from injury to arrival to the emergency room. Also, the zone of injury according to the Birmingham Eye Trauma Terminology Classification (BETT) and Ocular Trauma Classification (OTC) [5, 6] was analyzed. Moreover, presenting signs like corneal laceration, scleral laceration, hyphema, hypopyon, afferent pupillary defect, iris prolapse, cataract, vitreous hemorrhage, retinal detachment, choroidal detachment, and presence of intraocular foreign body was identified.

The data collection also documented any facial trauma, eyelid laceration, surgeries needed, the post-operative course (Amblyopia, astigmatism, corneal scar, cataract, retinal detachment, soft eyeball), and also the visual acuity.

The data were collected and stored physically in datasheet papers and virtually stored, coded, and analyzed using Statistical Package for the Social Sciences (SPSS) IBM Corp. Released 2019. IBM SPSS Statistics for Windows, Version 26.0. Armonk, NY: IBM Corp. Analysis was done and variables were presented as frequencies and percentages. A Chi-square test was performed and a p-value below < 0.05 was considered statistically significant. 

## Results

**Figure 1:**
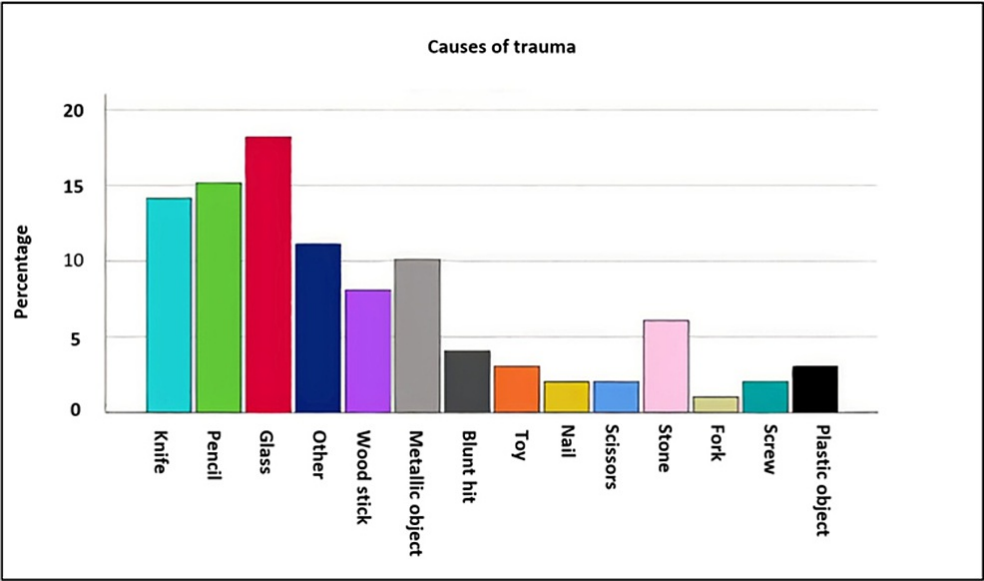
The objects causing the injury and its percentage.

Around 120 cases were identified within the pediatric age group ≤ 12 years. Eighty-two of the documented 118 (69.5%) patients were males and thirty-six (30.5%) were females. The mean age was 5.13 years. Out of the documented data in 101 cases, the mechanism of trauma was penetrating in 90 (89.1%) cases and blunt in 11 (10.9%) cases. The object causing the injury was documented in 98 cases in which the most common tool was glass, documented in 18 (18.4%) patients, a pencil was in 15 (15.3%), a knife was in 14 (14.3%) and 10 (10.2%) patients’ injuries were caused by other metallic objects (Figure 1). 

The most common object in age group 0 to four was glass followed by knife, while in five to eight age group, it was metallics. In the nine to 12 age group, the most common object was pencil (Table 1).

**Table 1:** The objects causing injury relative to age group.

Objects	Age Groups			
	0-4	5-8	9-12	Total
Knife	8	4	1	13
Pencil	4	3	6	13
Glass	10	6	2	18
Plastic object	0	2	0	2
Wood stick	2	2	2	6
Metallic object	3	7	0	10
Blunt hit	2	2	0	4
Toy	2	1	0	3
Nail	1	1	0	2
Scissor	1	0	1	2
Stone	0	2	4	6
Fork	1	0	0	1
Screw	1	0	1	2
Others	8	1	1	10

Most cases were accidental (68.9%) and 48 (71.6%) cases out of the documented 67 cases occurred inside the house. The duration between injury to arrival to the emergency room was less than 24 hours in 76 (67.3%) out of 113 cases. The presenting signs documented in 120 patients were corneal laceration which was noted in 98 (81.7%) cases, scleral laceration in 19 (15.8%) cases, hyphema in 23 (19.2%) cases. Additionally, iris prolapse was seen in 52 (43.3%) cases, traumatic cataract in 20 (16.7%) cases, vitreous hemorrhage in 10 (8.3%) cases, retinal detachment in six (5%) cases, and intraocular foreign body in 24 (20%) cases. Eighty-two (68.3%) patients underwent one operation, while 38 (31.7%) needed further operations.

The zone of injury was documented in 115 patients. Zone 1 injury was documented in 95 (82.9%) patients, zones 2 and 3 were in 19 (16%) and one (0.9%) patients, respectively. Eyelid lacerations were present in 11 (9.2%) patients, laceration elsewhere was in five (4.2%) patients, and facial trauma was in four (3.3%) patients.

Follow-up data was documented in 115 patients, the rest failed to follow up in the same hospital. Almost 34 (29.6%) patients developed cataract, 38 (33%) had corneal scar, four (3.5%) developed amblyopia, three (2.6%) developed astigmatism, and further three (2.6%) developed retinal detachment.

Factors that have a significant impact on ultimate visual acuity were the mechanism of injury, hyphema, vitreous hemorrhage, intraocular foreign body, the number of surgeries required, presence of cataract at presentation. The factors that were associated with better visual outcome (20/40 or better) are sharp injury, absence of hyphema, absence of vitreous hemorrhage, needing only one operation, absence of cataract at presentation, and surprisingly presence of the intraocular foreign body (Table 2).

**Table 2:** Factors predicting ultimate visual acuity of ⩾20/40 or better.

Variables	Total number	Category	Number	N (%) ⩾20/40 V/A	P-value
Age	70	0-4	28	11 (39.3%)	0.953
		5-8	26	11 (42.3%)	
		9-10	16	7 (43.8%)	
Gender	74	Male	53	20 (37.7%)	0.435
		Female	21	10 (47.6%)	
Mechanism of injury	61	Sharp	53	23 (43.4%)	0.018
		Blunt	8	0 (0%)	
Time of reporting to emergency	70	Within 24h	48	18 (37.5%)	0.324
		More than 24h	22	11 (50%)	
Corneal laceration	75	Yes	65	26 (40%)	0.550
		No	10	5 (50%)	
Hyphemia	75	Yes	16	2 (12.5%)	0.008
		No	59	29 (49.2%)	
Irises prolapse	75	Yes	35	11 (31.4%)	0.103
		No	40	20 (50%)	
Vitreous hemorrhage	75	Yes	7	0 (0%)	0.020
		No	68	31 (45.6%)	
Retinal detachment	75	Yes	2	0 (0%)	0.229
		No	73	31 (42.5%)	
Intraocular foreign body	75	Yes	14	10 (71.4%)	0.011
		No	61	21 (34.4%)	
Number of surgeries	75	one	46	28 (60.9%)	0.0001>
		>1	29	3 (10.3%)	
Cataract	75	Yes	24	4 (16.7%)	0.003
		No	51	27 (52.9%)	

## Discussion

This retrospective study provides an in-depth analysis of pediatric open globe injury in the study region. Most of the cases in the study were males, which is similar to previous studies [7-9]. This implies that males tend to be more engaged in risky behaviors. Studies with a similar background in Kuwait [10], and Turkey [11], have had similar results as the glass was the most frequent object causing injury, this was anticipated due to the similarity of the environments in these countries and Saudi Arabia. Other studies reported that knives were the most common object [7,12,13]. Around 20% of the current cases were found to have an intraocular foreign body which is higher than previous studies [7,9,13]. It could be attributed to shards from broken glass. It was documented that most cases had occurred inside the house, in contrast to a prior study from Turkey [13], which showed that playgrounds were more common to have traumatic open globe injuries. The majority of injuries as the current study revealed had occurred in zone 1 like most of the studies [7-9,13]. It was found that 38 (31.7%) cases required additional surgery after the primary repair of the open globe, mostly because of the formation of a traumatic cataract, vitreous hemorrhage, retinal repair, or enucleation.

In this study, it was identified that there was a significant association between the presenting signs and the visual outcome. The presence of hyphema, cataract and vitreous hemorrhage has a significant association with poorer visual outcomes, which is consistent with other studies [7,13,14]. Surprisingly, intraocular foreign body was significantly associated with better visual outcome (20/40 or better VA), while other studies found it to have an insignificant impact on visual outcome [7,9]. It was also observed that the majority of the patients with an intraocular foreign body in the current study had a mild injury. This could be altered if the sample size was larger.

Unlike the Turkish and Saudi studies [7,13], it was found that the iris prolapse had an insignificant association with visual outcome. The current finding is consistent with reports from Kuwait and Canada [10,14]. This might be related to the sample size. The current study showed blunt trauma had worse ultimate visual acuity, which contrasts with the Saudi study [7].

The current study might have underestimated the definite number of cases because only injuries presented to the tertiary hospitals were included. Furthermore, medical data was limited because it was obtained from patients’ medical records. Also, the sample size was inadequate to detect the impact of retinal detachment on ultimate visual acuity.

## Conclusions

The presence of hyphema, cataract, blunt trauma, and vitreous hemorrhage has a significant association with poorer visual outcomes. In contrast to other studies, we found that intraocular foreign body was significantly associated with better visual outcome, and iris prolapse has an insignificant association with visual outcome. Males were found to have higher rates of accidents and thereby are more prone to open globe injury because of the natural risky behaviors. Furthermore, the household environment carries the highest percentage of injuries. This should emphasize the role of parents and caregivers to decrease the incidence of ocular trauma by applying safety measures, which include childproofing the house and adult supervision of children.
